# Advances in Composite Stimuli-Responsive Hydrogels for Wound Healing: Mechanisms and Applications

**DOI:** 10.3390/gels11060420

**Published:** 2025-05-31

**Authors:** Ke Ding, Mingrui Liao, Yingyu Wang, Jian R. Lu

**Affiliations:** Biological Physics Group, Department of Physics and Astronomy, School of Natural Sciences, University of Manchester, Oxford Road, Manchester M13 9PL, UK; ke.ding@manchester.ac.uk (K.D.); mingrui.liao@manchester.ac.uk (M.L.); yingyu.wang@manchester.ac.uk (Y.W.)

**Keywords:** stimuli-responsive hydrogels, composite hydrogels, wound healing, bioactive agents, controlled drug release, antibacterial hydrogels, smart biomaterials, biomedical applications

## Abstract

Stimuli-responsive hydrogels have emerged as a promising class of biomaterials for advanced wound healing applications, offering dynamic and controllable responses to the wound microenvironment. These hydrogels are designed to respond to specific stimuli, such as pH, temperature, light, and enzyme activity, enabling precise regulation of drug release, antimicrobial activity, and tissue regeneration. Composite stimuli-responsive hydrogels, by integrating multiple response mechanisms and functions, show potential for addressing the diverse needs of wound healing. This review explores the biological mechanisms of wound healing, the design and classification of composite stimuli-responsive hydrogels, and the key fabrication strategies employed to optimise their properties. Despite their immense potential, unresolved challenges such as biocompatibility, long-term stability, and scalability continue to limit their translation into clinical practice. Future research will focus on integrating hydrogels with smart wearable devices, AI-driven personalised medicine, and 3D bioprinting technologies to develop next-generation wound care solutions. With continuous advancements in biomaterials science and bioengineering, stimuli-responsive hydrogels hold great promise for revolutionising wound management.

## 1. Introduction

Wound healing is a complex and highly regulated biological process that plays a crucial role in restoring skin integrity and function following injury [1,2]. This process involves four overlapping yet distinct phases—hemostasis, inflammation, proliferation, and remodelling—each governed by intricate cellular and molecular interactions [3]. These phases function in a coordinated manner to restore tissue integrity following injury, thereby facilitating proper wound closure and functional recovery [4]. However, in chronic wounds such as diabetic ulcers, pressure sores, and severe burns, the delicate balance of these phases is often disrupted due to persistent inflammation, bacterial infection, and impaired vascularisation, leading to delayed healing and poor clinical outcomes [5].

To address these challenges, various wound care strategies have been developed, ranging from traditional dressings to advanced biomaterials [6,7]. While conventional dressings, such as gauze, primarily serve as passive protective barriers, they lack the ability to actively modulate the wound microenvironment [8,9,10]. In contrast, stimuli-responsive hydrogels have emerged as intelligent biomaterials capable of dynamically adapting to physiological conditions [11,12]. These hydrogels show significant potential in accelerating wound healing, enhancing antimicrobial defence, and promoting tissue regeneration [13,14]. Designed to respond to specific physiological or external stimuli—such as pH, temperature, enzymes, light, or electrical signals—these hydrogels can release bioactive molecules or undergo controlled structural transformations [15,16].

**Figure 1:**
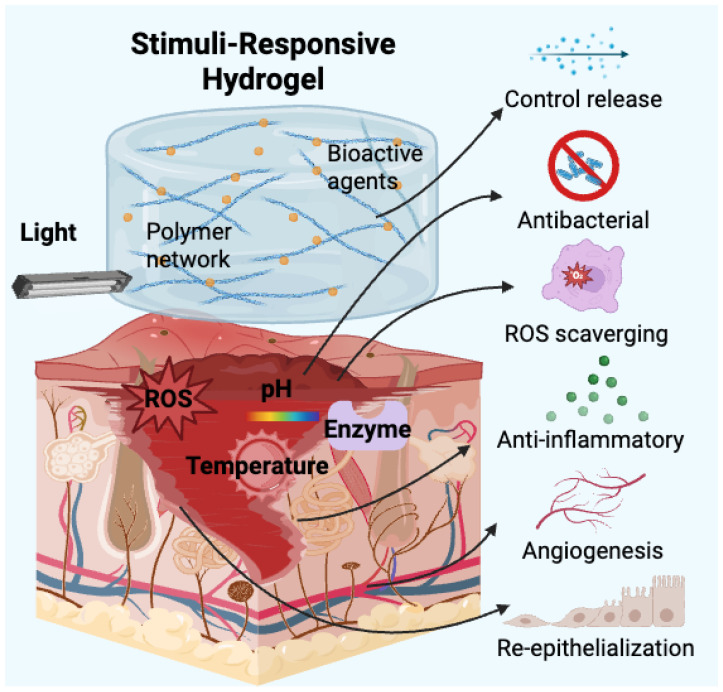
Schematic illustration of stimuli-responsive mechanisms and therapeutic functions of composite hydrogels in wound healing.

Compared with traditional wound dressings, stimuli-responsive hydrogels offer several distinct advantages [17]. Their high water content mimics the natural ECM, providing a moist environment that facilitates cell proliferation, migration, and differentiation [18,19]. Additionally, their biocompatibility, biodegradability, and tunable mechanical properties allow for customised designs tailored to different wound types [20]. More importantly, by integrating multiple responsive mechanisms, these hydrogels can function as intelligent drug delivery systems, enabling the targeted and on-demand release of therapeutic agents, such as antimicrobial peptides (AMPs), growth factors, and anti-inflammatory drugs [21]. This not only enhances treatment efficacy but also minimises systemic side effects [22]. Recent advances have further led to the development of composite stimuli-responsive hydrogels, incorporating nanomaterials, conductive polymers, bioactive molecules, and hybrid biomaterials, further broadening their therapeutic applications in wound management [23]. To provide a visual overview of the fundamental principles discussed, Figure 1 illustrates the core mechanisms by which composite stimuli-responsive hydrogels interact with wound environments to enable dynamic and targeted therapeutic effects.

Despite these promising developments, significant challenges remain in translating stimuli-responsive hydrogels from laboratory research to clinical practice [24]. Issues such as long-term stability, large-scale manufacturing, regulatory approval, and cost-effectiveness need to be addressed before these materials can achieve widespread adoption in clinical settings [25,26]. Nevertheless, the integration of multifunctional hydrogels with emerging technologies, such as wearable biosensors, real-time monitoring systems, and personalised medicine, is expected to revolutionise wound care in the near future [27,28].

This review provides a comprehensive analysis of the latest advances in composite stimuli-responsive hydrogels for wound healing. First, we discuss the biological mechanisms of wound healing, emphasising the key cellular and molecular players involved. Next, we explore the design strategies and classification of stimuli-responsive hydrogels, detailing their material composition, functional properties, and stimulus-triggered responses. Special emphasis is placed on functionalised hydrogels with active substance loading, highlighting their antibacterial, anti-inflammatory, and regenerative capabilities. Finally, we examine current clinical applications, existing challenges, and future research directions in this rapidly evolving field. In summarising recent breakthroughs and identifying key obstacles, this review aims to provide valuable information on the development of next-generation smart hydrogel-based wound dressings with enhanced therapeutic potential.

## 2. Biological Mechanisms of Wound Healing

**Figure 2:**
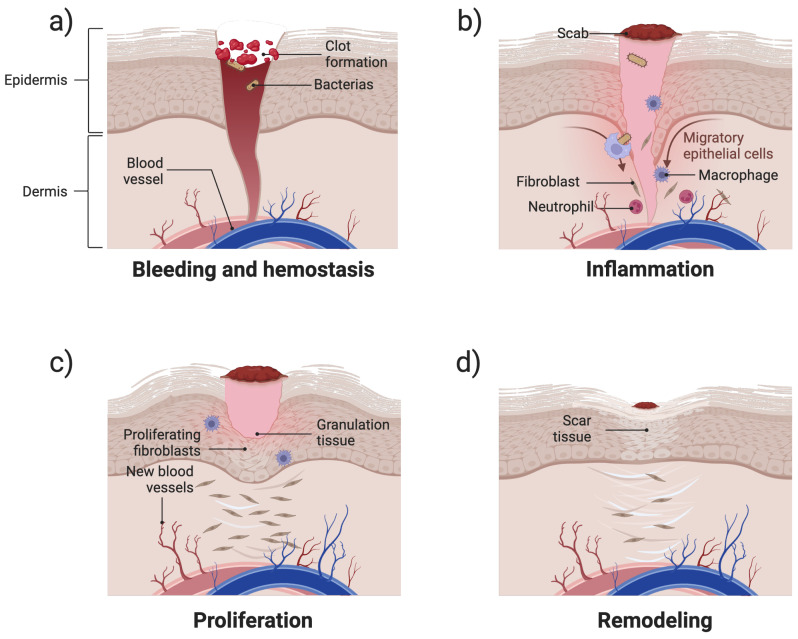
Schematic illustration of the four stages of wound healing: (**a**) hemostasis—blood clot formation and vessel constriction; (**b**) inflammation—infiltration of immune cells for pathogen clearance; (**c**) proliferation—fibroblast activation, collagen deposition, angiogenesis, and re-epithelialisation; and (**d**) remodelling—collagen remodelling and scar tissue formation.

Wound healing is a dynamic and intricate biological process composed of a series of overlapping yet distinct phases [29]. This highly regulated process involves complex interactions among coagulation factors, growth factors, cytokines, and components of connective tissue and vascular networks [30]. As shown in Figure 2a, the hemostasis phase starts immediately after injury, where platelets aggregate and release clotting factors to form a fibrin network that prevents excessive bleeding [31]. This is followed by an inflammatory response (Figure 2b), in which neutrophils and macrophages infiltrate the wound site to eliminate pathogens and clear cellular debris [13,32]. Subsequently, during the proliferation phase, fibroblasts deposit extracellular matrix (ECM) components, keratinocytes contribute to re-epithelialisation, and endothelial cells promote angiogenesis, all of which are essential for tissue regeneration [32,33] (Figure 2c). Finally, the remodelling phase strengthens newly formed tissue through collagen synthesis, crosslinking, and reorganisation, ensuring proper wound closure and functional recovery [34] (Figure 2d). Based on their pathophysiological mechanisms and clinical outcomes, wounds can be classified as acute or chronic [35,36]. The fundamental distinction between these categories lies in the biochemical microenvironment at the wound site and the concentration of specific bioactive components [37].

Acute wounds usually result from surgical incisions, trauma, or burns and progress through the normal wound healing stages in a predictable timeframe, usually completing the repair process in a few weeks [35,38]. These wounds exhibit a well-regulated inflammatory response, in which immune cells effectively remove pathogens and necrotic debris, allowing the subsequent proliferation and remodelling phases to proceed without significant interruption [39]. Growth factors, such as platelet-derived growth factor, transforming growth factor β, and vascular endothelial growth factor (VEGF), play a key role in stimulating fibroblast migration, collagen synthesis, and angiogenesis [40]. As a result, acute wounds generally heal with minimal scarring and speedy functional restoration of the affected tissue [41].

In contrast, chronic wounds such as diabetic foot ulcers and infected wounds often fail to progress through the typical wound healing cascade, remaining stalled in the inflammatory phase and resulting in impaired tissue regeneration [42,43]. A major contributing factor is persistent infection or impaired immune regulation, which disrupts the balance between inflammation and tissue regeneration [44]. This imbalance results in a sustained elevation of inflammatory cytokines such as tumour necrosis factor-alpha (TNF-α) and interleukin-6 (IL-6), alongside the suppressed expression of essential growth factors like VEGF and epidermal growth factor (EGF), ultimately impairing fibroblast activity and angiogenesis [45,46]. Moreover, overexpression of matrix metalloproteinases (MMPs) leads to excessive degradation of extracellular matrix (ECM) components, further hindering tissue regeneration [47].

Among chronic wounds, diabetic ulcers represent a particularly challenging subtype. Chronic hyperglycemia impairs endothelial function, delays angiogenesis, and contributes to microvascular dysfunction. It also alters neutrophil and macrophage behaviour, resulting in impaired immune responses. Diabetic wounds are often characterised by poor granulation tissue formation, reduced collagen deposition, and diminished keratinocyte migration, all of which contribute to delayed or incomplete re-epithelialisation [45]. Additionally, the accumulation of advanced glycation end-products and elevated oxidative stress further aggravate tissue damage, while peripheral neuropathy and ischemia impair wound sensation, perfusion, and repair capacity [47]. Stimuli-responsive hydrogels designed for diabetic wound healing often incorporate ROS scavenging mechanisms and enzyme-sensitive degradation pathways to mitigate oxidative stress and enable on-demand drug release. For example, a supramolecular hydrogel system composed of a hexapeptide containing methionine residues exhibits both ROS-scavenging capacity and ROS-responsive degradation. Upon exposure to elevated ROS levels, the Met residues are oxidised, triggering hydrogel disassembly and subsequent release of encapsulated therapeutics or bioactive molecules. This degradation process not only facilitates controlled release but also actively reduces local oxidative stress [48].

Wound infection is another major complication that can significantly impair healing and increase tissue damage, particularly in chronic wounds [49]. Burn injuries may initially be acute but often progress to chronicity, as rapid microbial colonisation and loss of skin barrier function can increase the risk of infection and impaired healing [49]. Upon bacterial invasion, pathogens can form biofilms: complex microbial communities embedded in a self-produced ECM that protects them from immune clearance and antimicrobial agents [50]. These biofilms prolong inflammation, elevate oxidative stress, and release toxins and proteolytic enzymes that contribute to tissue degradation. Components of the native ECM, such as fibronectin, fibrinogen, and collagen, serve as adhesion sites for biofilm formation [50,51]. Additionally, high levels of reactive oxygen species (ROS) and prolonged neutrophil activity induce oxidative damage to cellular structures, impairing keratinocyte migration and fibroblast function [52]. Infected wounds particularly benefit from stimuli-responsive hydrogels that offer controlled antibacterial agent release or physical antibacterial mechanisms. pH-responsive hydrogels can detect alkaline shifts associated with bacterial infection and subsequently release antimicrobial agents in a site-specific manner [53]. In addition, photothermal agent-integrated light-responsive or enzyme-degradable hydrogels have demonstrated potent biofilm-disrupting activity while minimising systemic toxicity [54,55].

Overall, the persistence of inflammatory mediators, excessive proteolysis, and bacterial colonisation collectively contribute to delayed wound closure and increased risks of tissue necrosis and systemic infection [56,57]. These pathophysiological variations across wound types, ranging from acute surgical wounds to chronic diabetic, infected, or burn wounds, highlight the need for tailored therapeutic strategies. Addressing these challenges requires targeted interventions that can regulate inflammation, support cellular activity, and promote ECM remodelling, areas in which stimuli-responsive hydrogel-based dressings show significant promise [58,59].

## 3. Classification Based on Stimuli Types

Compared with conventional hydrogels, which serve primarily as passive moisture-retaining or drug-releasing materials, stimuli-responsive hydrogels are capable of undergoing reversible or irreversible physical and/or chemical changes in response to specific environmental cues such as pH, temperature, ROS, enzymes, or light [58]. These systems offer dynamic, microenvironment-adaptive behaviours that are particularly valuable in wound healing applications. The microenvironment of chronic and infected wounds is highly heterogeneous, featuring fluctuating pH, oxidative stress, enzymatic activity, and bacterial burden. Stimuli-responsive hydrogels are uniquely suited to such conditions, enabling localised controlled therapeutic release, real-time responsiveness, and integration with biosensing or regenerative cues. A summary of these stimuli, their response mechanisms, therapeutic benefits, and typical application features in wound healing is presented in Table 1. In this section, we first discuss hydrogels that respond to a single type of stimulus, followed by composite systems designed to respond to multiple stimuli simultaneously.

### 3.1. Single Stimuli-Responsive Mode

#### 3.1.1. pH-Responsive Hydrogels

Changes in pH can affect inflammation, cell proliferation, bacterial growth and oxidative stress during the wound healing process, thereby directly impacting the speed and quality of wound healing [16,60]. Under normal conditions, the skin maintains a mildly acidic pH ranging from 5 to 6.5, which supports the skin barrier function and inhibits pathogenic microorganisms. However, when the skin is damaged, the wound environment undergoes pH alterations that influence the healing process [61,62]. Acute wounds typically exhibit a pH between 6.0 and 6.5, whereas chronic wounds often become more alkaline, with pH levels exceeding 7.3 [63,64]. pH-responsive hydrogels have emerged as promising materials for enabling controlled drug release and monitoring healing progress [11,65].

**Figure 3:**
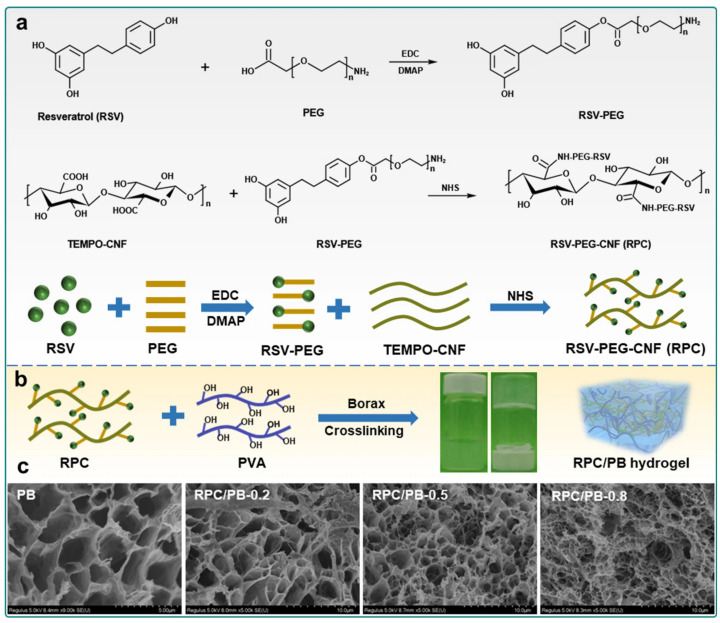
Schematic illustration of the preparation of pH-responsive RPC/PB hydrogel fabricated from PB and RPC: (**a**) synthetic route for the preparation of the RPC conjugate, (**b**) schematic illustration of the fabrication process of RPC/PB hydrogels, and (**c**) SEM images of PB hydrogel and RPC/PB hydrogels with varying RPC content. Reproduced from [72], licensed under CC BY 4.0.

Polyelectrolyte-based hydrogels rely on ionisable functional groups, such as carboxyl (-COOH), amino (-NH_2_), and sulphonic acid (-SO_3_H), which undergo protonation or deprotonation depending on the pH environment [66,67]. This process regulates the swelling behaviour of the hydrogel and influences drug release [68]. In acidic conditions, carboxyl groups remain protonated (-COOH), leading to hydrogel contraction, whereas in alkaline conditions, they deprotonate (-COO^−^), causing the hydrogel to swell [69]. Similarly, amino groups (-NH_2_) become protonated (-NH_3_^+^) in acidic environments, further affecting the hydrogel’s responsiveness [70]. These properties make polyelectrolyte-based hydrogels highly effective for controlled drug delivery, particularly in pH-sensitive environments such as inflamed or infected tissues [71]. A notable example is the pH-responsive semi-interpenetrating network (semi-IPN) hydrogel composed of resveratrol (RSV)-grafted cellulose nanofibrils (CNFs) and poly(vinyl alcohol)–borax (PVA–borax, PB), hereafter referred to as RPC/PB hydrogel, as shown in Figure 3. In this design, RSV-grafted CNF (abbreviated as RPC) reinforces the PVA–borax matrix, enabling enhanced mechanical strength and functionality. The hydrogel demonstrated a pH-dependent drug release profile, with RSV release at pH 5.4 being 2.33 times higher than that at pH 7.4, effectively adapting to the acidic wound environment. Furthermore, the RPC/PB hydrogel exhibited excellent antibacterial activity, biocompatibility, and antioxidant capacity [72].

Dynamic covalent bond-based hydrogels, on the other hand, utilise pH-sensitive reversible covalent bonds, such as hydrazone, imine (Schiff base), and oxime bonds, which can form or break in response to pH changes [73,74]. These bonds provide hydrogels with self-healing, injectable, and degradable properties [75]. Hydrazone bonds, formed between aldehyde (-CHO) and hydrazide (-NH-NH_2_) groups, are particularly sensitive to acidic conditions, facilitating controlled degradation [67]. Imine bonds, resulting from aldehyde and amine (-NH_2_) reactions, are more stable in alkaline environments but degrade under acidic conditions [76]. This class of hydrogels is widely used in tissue engineering, wound healing, and biomedical adhesives due to their ability to dynamically respond to physiological pH variations [77]. For instance, hyaluronic acid–aldehyde (HA–ALD) forms pH-responsive hydrazone bonds with adipic dihydrazide (ADH) and imine bonds with N-carboxyethyl chitosan (N-CS), resulting in a self-healing, injectable, and pH-responsive hydrogel. The decrease in pH accelerates the degradation of the hydrogel. Due to the presence of acylhydrazone bonds, lowering the pH of the medium also promotes the release of insulin from the hydrogel. The incorporated insulin glargine is continuously released from the hydrogel and remains pH-responsive for up to 14 days [78]. Similarly, Suo et al. developed a pH-responsive hydrogel based on hyaluronic acid (HA), using an AMP (KK(SLKL)_3_KK) as a crosslinker through Schiff base formation. This hydrogel releases AMP in response to acidic conditions and exhibits excellent broad-spectrum antibacterial activity [79]. Furthermore, He et al. [80] developed a series of injectable, pH-responsive, self-healing adhesive hydrogels based on acryloyl-6-aminohexanoic acid (AA) and AA-g-N-hydroxysuccinimide (AA-NHS). AA-NHS acts as a micro-crosslinker and forms dynamic covalent bonds with amines on bacterial membranes, leading to strong adhesion. These hydrogels have demonstrated excellent therapeutic effects and significantly enhanced wound healing.

Beyond drug delivery, pH-responsive hydrogels also hold great promise in wound infection monitoring and biosensing applications [61]. Since wound pH changes markedly in response to bacterial colonisation and inflammation, these hydrogels can function as smart diagnostic tools that respond to local microenvironmental cues [67]. For example, hydrogels embedded with pH-sensitive dyes can undergo visible colour changes, depending on the local pH. Huang et al. developed an injectable hydrogel incorporating anthocyanins as a natural pH-sensitive dye, enabling visible pH monitoring within the range of 5 to 9. The hydrogel exhibited a distinct colour transition from purplish–red to bluish–green, effectively identifying wound infection [81]. This provides a non-invasive visual cue for detecting infection or delayed healing. Such indicators are particularly valuable for chronic wounds in elderly or diabetic patients, enabling early intervention without the need for laboratory analysis [82]. In addition, some systems combine pH sensitivity with electronic sensing. For instance, conductive pH-sensitive hydrogels integrated with flexible electrodes can convert local pH fluctuations into electrical signals for real-time digital monitoring [61]. Knopf and Sinar immobilised a pH-sensitive chitosan hydrogel onto interdigitated capacitive electrodes, where pH-induced swelling or shrinkage of the hydrogel caused measurable changes in circuit capacitance [83]. These approaches highlight the dual functionality of pH-responsive hydrogels as both therapeutic carriers and wound-responsive biosensors [82].

#### 3.1.2. Temperature-Responsive Hydrogels

Temperature plays a crucial role in wound healing as it can act as a trigger or response signal for specialised wound dressings [84]. Thermoresponsive hydrogels hold great potential in drug or cell delivery systems and damaged tissue repair. Due to their controllability, they are commonly used in the design of responsive systems [85,86].

Thermosensitive hydrogels undergo a reversible sol–gel phase transition within a specific temperature range, making them highly valuable for wound healing applications [86,87]. These hydrogels serve as drug carriers, enabling controlled release while maintaining a moist wound environment that promotes cell proliferation and tissue regeneration [88]. The phase transition behaviour of thermosensitive hydrogels is typically governed by the lower critical solution temperature (LCST) or the upper critical solution temperature (UCST) [89]. LCST-type hydrogels gelate when heated above their LCST, whereas UCST-type hydrogels undergo gelation when cooled below their UCST [90]. This temperature-responsive property makes thermosensitive hydrogels highly promising for wound care, antibacterial therapy, and skin regeneration [91].

LCST hydrogels undergo gelation when the temperature rises above LCST due to the hydrophilic-to-hydrophobic transition within the polymer structure [92,93]. For instance, poly(N-isopropylacrylamide) (PNIPAM) is a typical LCST polymer that remains hydrated in a linear chain structure below its LCST but aggregates into a gel above its LCST due to enhanced hydrophobic interactions [94]. PNIPAM and its modified hydrogels have been extensively utilised in wound healing due to their excellent thermosensitivity and biocompatibility [95]. For example, a multifunctional collagen-based hydrogel (COL-GG-PNIPAM) was developed, incorporating PNIPAM to endow it with excellent thermoresponsiveness and near-infrared (NIR) responsiveness, demonstrating a significantly enhanced wound healing effect (81.0 %) in mouse skin injury repair [96]. Furthermore, Feng et al. [97] developed a multifunctional composite hydrogel based on a PNIPAM polymer (PNI/RA-Amps). This hydrogel undergoes a phase transition at 32 °C and rapidly gels, significantly enhancing wound healing by promoting epithelialisation, neovascularisation, and collagen fibre formation. A series of thermoresponsive graft copolymers based on an alginate backbone and P(NIPAMx-co-NtBAMy) side chains were designed, forming self-assembling hydrogels through hydrophobic interactions, with the gelation temperature tunable from 38 °C to 20 °C by varying NtBAM content, enabling shear-thinning injectability at room temperature and rapid stiffening at body temperature [98]. Apart from synthetic polymers, natural LCST polymers such as methylcellulose [99] and xanthan gum [100] also exhibit thermosensitive gelation behaviour [89].

UCST hydrogels gelate when the temperature drops below their UCST, a process primarily driven by the transition of polymer chains from a random coil state at high temperatures to an ordered helical structure at lower temperatures [90,91]. For example, agarose–gelatin–carboxymethyl cellulose ternary bioinks have been used for 3D-printing personalised wound dressings, where their thermosensitive properties allow easy shaping at low temperatures while facilitating gradual release of bioactive factors at body temperature [101]. Additionally, the agarose–chitosan composite hydrogel undergoes in situ gelation as the temperature decreases from 50 °C to 37 °C. The resulting hydrogel dressing has been proven to possess antibacterial properties, effectively promoting tissue growth and accelerating wound healing [91].

In addition to controlled drug delivery, thermoresponsive hydrogels have shown great promise in activating mechanotransduction pathways that facilitate wound healing [102,103]. These hydrogels undergo a reversible volume phase transition at physiological or pathological temperatures, resulting in shrinkage or swelling of the matrix [104]. This physical deformation generates compressive or tensile forces on the surrounding tissues and cells, thereby modulating cellular behaviour through mechanical cues [86,102]. Zhao et al. [105] developed a thermoresponsive interpenetrating network hydrogel (PNI-HA) that contracts upon body temperature, generating mechanical tension at the wound interface, which activates mechanotransduction signalling and significantly accelerates wound closure in mice. Similarly, Li et al. [106] designed a thermocontractile hydrogel that promotes basal cell proliferation and tissue regeneration by triggering YAP nuclear translocation and concurrent MEK pathway activation. Both YAP/TAZ and MEK signalling processes are known to play key roles in re-epithelialisation and matrix remodelling [107]. Therefore, beyond acting as passive carriers, thermoresponsive hydrogels can function as active mechanical microenvironments to dynamically stimulate tissue regeneration.

#### 3.1.3. Enzyme-Responsive Hydrogels

Enzyme-responsive hydrogels are a class of smart biomaterials capable of undergoing structural or functional changes upon exposure to specific enzymes [108]. These hydrogels typically incorporate crosslinkers or functional groups that can be recognised and degraded by enzymes, leading to modifications in their swelling, degradation, or dissolution behaviour [109]. Due to their ability to respond to enzymatic activity, these hydrogels have been widely explored for applications in drug delivery and wound healing [110]. The primary mechanisms of enzyme responsiveness include enzyme-catalysed degradation, enzyme-induced swelling, or contraction [111].

**Figure 4:**
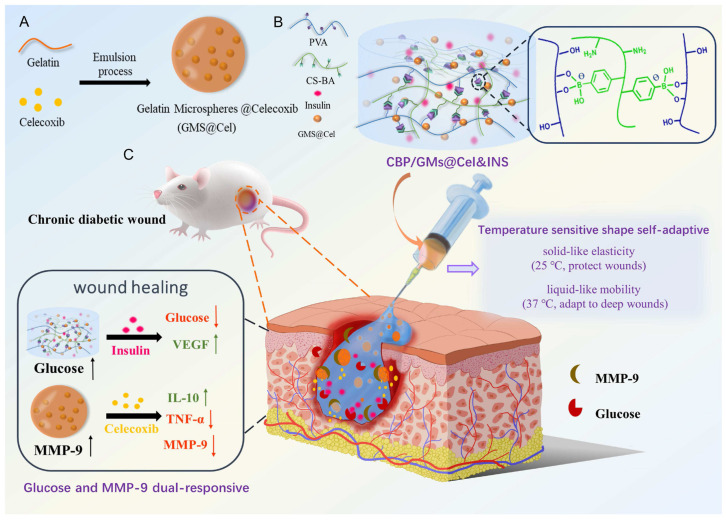
Glucose and MMP-9 dual-responsive, shape-self-adaptive hydrogels for chronic diabetic wound treatment: (**A**) preparation of GMs@Cel (celecoxib-loaded gelatin microspheres), (**B**) fabrication and characteristics of CBP/GMs@Cel&INS hydrogel, and (**C**) treatment of chronic diabetic wounds with CBP/GMs@Cel&INS hydrogel via dual-responsive system. Reproduced from [117] with permission from Elsevier.

Enzyme-catalysed degradation is one of the most common mechanisms in enzyme-responsive hydrogels [112]. Specific enzymes can recognise and hydrolyse chemical bonds within the hydrogel network, leading to its breakdown and the controlled release of encapsulated drugs [60]. For instance, a silver nanocluster (Ag NC) composite hydrogel was designed to respond to bacterial enzymes, including proteases and DNase, which are overexpressed in infected wounds [113]. These enzymes degrade the hydrogel matrix, triggering the controlled release of antibacterial silver ions [113]. The hydrogel exhibited strong antibacterial effects against *Staphylococcus aureus* and *Escherichia coli*, significantly promoting cell proliferation and wound healing while reducing inflammation [113]. Distler et al. [114] reported a dual-crosslinked hydrogel system based on aldehyde-functionalised alginate (ADA) and gelatin (GEL) (ADA-GEL), which is crosslinked via ionic interactions with Ca^2+^ and enzymatic reactions using microbial transglutaminase (mTG). By adjusting the concentration of mTG in the crosslinking solution, the degradation rate of the hydrogel could be tuned from fast (<7 days) to moderate (14 days) and slow (>30 days) [114]. Similarly, adipose-derived stem cell exosomes (ADSC-exo) were loaded into a matrix metalloproteinase (MMP)-degradable polyethylene glycol (PEG) hydrogel [115]. As the hydrogel degrades in response to MMP activity, ADSC-exo is gradually released, enhancing cell function through Akt signalling, reducing H_2_O_2_-induced oxidative stress, and promoting diabetic wound healing [115]. To further enhance wound healing, Li et al. [116] designed an MMP-responsive hydrogel loaded with deferoxamine (DFO) to promote angiogenesis by increasing the expression of hypoxia-inducible factor-1α (HIF-1α). The MMP-degradable hydrogel enables the sustained release of DFO over 24 h, addressing its short half-life and potential neurotoxicity [116]. Additionally, a glucose and MMP-9-responsive, temperature-sensitive, shape-adaptive hydrogel was developed for the on-demand release of insulin and celecoxib in high-glucose and MMP-9-rich environments, as shown in Figure 4. This hydrogel exhibited remodelling and self-healing properties, significantly reducing inflammation, regulating local high glucose and MMP-9 levels, and effectively promoting wound healing [117].

Compared with traditional drug delivery systems, enzyme-triggered hydrogel degradation allows for an environment-responsive release profile, enabling precise control over drug administration in response to disease-specific enzymatic activity, such as inflammation or infection [118]. Future research in this field should focus on improving hydrogel stability, optimising enzyme-specific responsiveness, and expanding their applications in personalised medicine [119]. By integrating advanced biomaterial design with a deeper understanding of enzymatic interactions, enzyme-responsive hydrogels hold great potential for next-generation biomedical technologies [60].

#### 3.1.4. Photoresponsive Hydrogels

Photoresponsive hydrogels can change their physical and chemical properties in response to specific wavelengths of light [120]. These changes may include swelling, shrinking, or degradation. Such hydrogels typically contain light-sensitive functional groups or nanomaterials, allowing them to respond to ultraviolet (UV), visible (Vis), or NIR light [121]. Phototherapy mainly includes photodynamic therapy (PDT) and photothermal therapy (PTT), both of which use light to stimulate cellular processes. This approach helps prevent the development of multi-drug-resistant bacteria caused by excessive antibiotic use.

PDT utilises low-intensity visible light (VL) or NIR light to activate a photosensitiser, generating cytotoxic substances to kill target cells. Ag nanoparticles were incorporated into a photoresponsive metal–organic framework (MOF) hydrogel, enabling the photocatalytic generation of ROS under VL [122]. The boronic acid groups provided attachment sites for target bacteria and facilitated ROS transfer. Adding the incorporation of the cationic natural drug berberine enhanced the photodynamic antibacterial effect, significantly reducing inflammation and promoting wound healing [122].

In PTT, photothermal agents absorb photon energy and convert it into heat, raising the local temperature to selectively destroy abnormal cells. Liu et al. [123] innovatively constructed a CS/AM NS hydrogel by integrating antimonene nanosheets (AM NSs) with excellent photothermal properties and chitosan (CS). Under NIR laser irradiation, AM NSs efficiently convert light energy into localised heat, helping to eliminate bacteria.

Beyond individual applications of PDT and PTT, recent studies have focused on their synergistic integration to achieve enhanced therapeutic efficacy. A composite sprayable hydrogel was designed by Liu et al. [124] with a seamlessly integrated Bi/MoS_2_ nano-heterojunction. Leveraging the synergistic effects of PDT and PTT, just 10 min of 808 nm NIR irradiation achieved strong antibacterial effects. This accelerated both in vitro and in vivo bacterial elimination, promoting faster wound healing.

#### 3.1.5. ROS-Scavenging Hydrogels

ROS are produced during cellular metabolism and are critical to cellular processes [125]. Low levels of ROS favour normal wound healing by stimulating cell migration and angiogenesis [126]. However, excessive levels of ROS can impede or even jeopardise wound healing, especially in chronic wounds [127]. To accelerate chronic wound healing, many hydrogel dressings with antioxidant properties have been designed, aiming to neutralise ROS and reduce oxidative stress in tissues [125].

**Figure 5:**
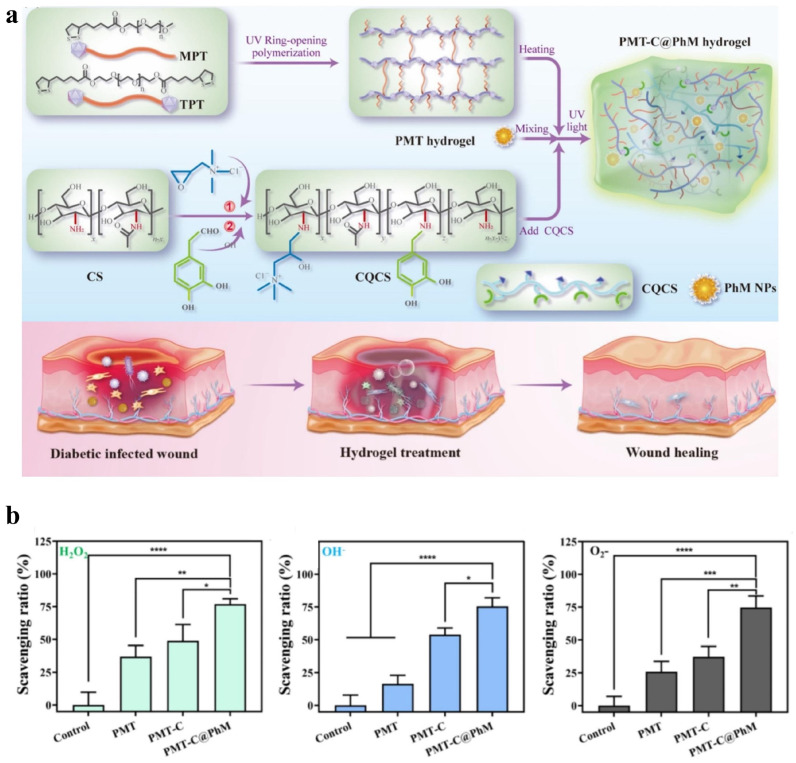
(**a**) Schematic diagram of the fabrication of PMT-C@PhM hydrogel for wound healing. (**b**) ROS (H_2_O_2_, OH^−^, $O_{2}^{-}$) scavenging efficiency of the hydrogels (n = 3). Statistical significance is indicated as follows: * p<0.05, ** p<0.01, *** p<0.001, **** p<0.0001. Adapted from [128] with permission from Elsevier.

Catalytic nanoparticles or functional groups with ROS scavenging ability are usually embedded in hydrogel matrices. For example, Wang et al. [128] incorporated quaternised and catechol-modified CS (CQCS) and polydopamine(PDA)-coated manganese dioxide nanoparticles (hMnO_2_@PDA NPs) into a lipoic acid-functionalised PEG network to create a CS-based biomass hydrogel (PMT-C@PhM) (Figure 5a). When exposed to hydrogen peroxide (H_2_O_2_), PMT-C@PhM showed the highest reactive oxygen species (ROS) scavenging efficiency (Figure 5b). This was attributed to the enzyme-like properties of manganese dioxide (MnO_2_) with mixed oxidation states and the antioxidant capability of PDA, which worked together to enhance the hydrogel’s ability to eliminate reactive nitrogen and oxygen species (RNOS). Pan et al. [129] embedded the antioxidant groups gallic acid (GA) and 3-aminophenylboronic acid (PBA) into a HA hydrogel, forming a dual-functional HA (HA-GA-PBA). This hydrogel exhibited antioxidant properties and strong tissue adhesion, promoting corneal epithelial wound healing. The results showed that the hydrogel increased the proliferation rate of human corneal epithelial cells by more than 2.5 times.

Another category involves hydrogels with ROS-cleavable crosslinkers [130]. These hydrogels incorporate ROS-responsive dynamic covalent bonds (such as thioether, selenoether, or enol ether bonds) into the polymer chains [125]. In oxidative environments, these crosslinkers break down, regulating hydrogel degradation, drug release, or mechanical properties to alleviate oxidative stress and promote tissue repair [126].

To provide a comprehensive overview of current design strategies, Table 2 summarises representative stimuli-responsive hydrogels developed for various types of wound healing, including acute, chronic, diabetic, infected, and burn wounds. These hydrogels respond to specific pathological cues such as pH, temperature, light, ROS, and enzymatic activity. They are constructed from a wide range of natural and synthetic polymers, including hyaluronic acid, chitosan, PNIPAM, and peptide-based materials, and function via mechanisms such as dynamic covalent bonding, sol–gel transitions, photothermal conversion, and ROS modulation. The designs of these systems offer a broad perspective on material selection, functional integration, and application potential, reflecting the trend toward engineering intelligent and adaptive hydrogels tailored to complex wound microenvironments.

### 3.2. Multi-Stimuli Responsive Hydrogels

Multi-stimuli responsive hydrogels react to multiple environmental triggers, such as pH, temperature, redox conditions, ROS, enzymes, and light [120]. By integrating multiple responsive mechanisms, these hydrogels can dynamically adjust their properties, such as swelling, degradation, and drug release, to suit specific biomedical applications [100].

For example, Wu et al. [149] developed a pH/ROS dual-responsive injectable glycopeptide hydrogel based on PBA-g-ODex and CA-g-ε-PL, exhibiting antibacterial and antioxidant properties. The hydrogel was loaded with mangiferin and the anti-inflammatory drug diclofenac sodium, with controlled release via pH-responsive micelles. The hydrogel relies on a pH/ROS-triggered degradation mechanism. At pH > pKa (7.8–8.6), PBA forms boronate ester bonds, maintaining hydrogel stability. In acidic and high-ROS environments, these bonds hydrolyse and selectively react with ROS, leading to hydrogel degradation and drug release. Meanwhile, Schiff base bonds hydrolyse under acidic conditions, further accelerating degradation. In response to intelligently responding to the inflammatory microenvironment, the hydrogel enables controlled drug release, promoting wound healing and demonstrating great biomedical potential. Zhang et al. [150] developed a dual-responsive pH / temperature composite hydrogel based on bamboo cellulose and in situ crosslinked carboxylated-β-cyclodextrin (BPCH-B), loaded with berberine. This hydrogel exhibited antibacterial activity, biocompatibility, and drug delivery capabilities. Experiments showed that drug release significantly increased (>70%) under alkaline pH (7.6) and high temperature (40 °C). Full-thickness wound healing experiments demonstrated that the BPCH-B group achieved 80% wound closure within 12 days, significantly outperforming the control group (50%). Tian et al. [151] developed a dual responsive photothermal/ROS smart hydrogel (CNMs@HA-PDA-PL) based on dopamine (DA)-modified graphitic carbon nitride microspheres (CNMs), DA-grafted AMPs (DA-PL) and DA polymerisation. The hydrogel facilitates infected wound healing through synergistic antibacterial activity and immune modulation. Under NIR irradiation, the photothermal-responsive PDA framework generates heat for PTT, effectively eliminating bacteria and reducing inflammation. Subsequently, hyaluronidase (HAase) overexpressed in the inflammatory microenvironment degrades HA fragments, releasing CNMs, which generate ROS under visible light irradiation, enabling broad-spectrum antibacterial disinfection. The synergistic effects of PTT and PDT rapidly eradicate bacteria and significantly suppress inflammation. Furthermore, hydrogen peroxide (H_2_O_2_), overexpressed in inflamed tissues, triggers the sustained release of ε-PL, further eliminating deep-seated bacteria and enabling long-term controlled inflammation regulation. During this process, M1 macrophages gradually transition to M2, promoting tissue regeneration and accelerating wound healing.

Multi-stimuli responsive hydrogels have emerged as promising biomaterials for precision medicine, particularly in wound healing [120]. Their ability to dynamically adapt to physiological changes enables more effective and controlled therapeutic strategies [42]. Future research may focus on improving hydrogel stability, improving response sensitivity, and developing novel biomimetic designs to optimise clinical applications [59]. In addition, integrating multifunctional nanomaterials and exploring patient-specific hydrogel formulations could further advance their potential in personalised medicine [100].

## 4. Conclusions

Stimuli-responsive hydrogels are a significant advancement in wound care, combining biocompatibility, controlled drug release, and the ability to adapt to changing conditions of wound environments. Their ability to manage infection, inflammation, and tissue regeneration provides clear benefits over traditional wound dressings. Recent developments have shown that multifunctional hydrogels, incorporating nanoparticles, bioactive agents, and responsive elements, can provide combined antibacterial, anti-inflammatory, and regenerative effects. These hydrogels respond to changes in the wound environment, such as oxidative stress, pH levels, and enzymatic activity, allowing precise, on-demand drug release. Hybrid hydrogels made from both natural and synthetic polymers also show promise in improving both mechanical properties and biological activity, making them suitable for use in a variety of wound types. However, most clinically intelligent hydrogel systems remain in early developmental stages, underscoring the need for continued efforts toward real-world applications.

## 5. Clinical Translation and Future Perspectives

Despite recent advances, significant challenges remain in scaling up production, reducing manufacturing costs, and navigating complex regulatory pathways. While several advanced wound dressings—such as Tegaderm^TM^ Hydrocolloid Thin Dressing, which maintains an occlusive moist environment, and silver ion-releasing hydrofibre dressings like AQUACEL^®^ Ag+ dressings, which provide sustained antimicrobial activity, have been commercially available for many years, but most stimuli-responsive hydrogels capable of reacting to specific physiological cues remain in the preclinical or early clinical stages of development. For example, the GAT@F nanozyme hydrogel dressing is currently undergoing a phase II clinical trial for diabetic wound healing (NCT06492811). Similarly, 5-aminolevulinic acid photodynamic therapy combined with hydrogel dressing is being investigated in an ongoing early-phase trial for infected chronic wounds (NCT06445699). These examples underscore the growing translational commitment and undertaking in smart hydrogel technologies, while also reflecting their nascent status in the clinical pipeline. Encouragingly, such trials indicate that the clinical translation of intelligent hydrogel systems is beginning to take shape [152].

Addressing the translational barriers of stimuli-responsive hydrogels requires a multifaceted strategy. From a manufacturing standpoint, improving crosslinking chemistries and standardising fabrication protocols will be essential to ensure batch-to-batch consistency and cost-effectiveness [153]. Integrating regulatory considerations early in the design process, e.g., selecting clinically validated polymers and adopting GMP-compatible procedures, can streamline approval pathways [14]. Mechanical reliability, stability in physiological fluids, and compatibility with clinical workflows should also be prioritised to enhance user acceptance and practical deployability. Equally critical is the long-term biocompatibility of hydrogel systems [154]. Future studies should focus on in vivo degradation profiling, chronic immune response monitoring, and cytotoxicity testing using standardised human-derived models [155]. These data will be essential to support regulatory approval and clinical confidence.

Looking forward, the clinical success of composite hydrogels will depend on their ability to combine therapeutic precision with functional versatility. One promising direction is the modular design of hydrogels that integrate antibacterial, anti-inflammatory, and pro-regenerative functionalities. Systems responsive to multiple stimuli such as pH, ROS, and enzymes are particularly suited to address the complex pathophysiology of chronic or infected wounds. Another key opportunity lies in integrating real-time sensing and responsive feedback. Hydrogels embedded with biosensors to detect changes in pH, glucose, or oxidative stress may enable adaptive, personalised therapy [156]. Coupling these sensing functions with controlled drug-release modules or digital wound monitoring platforms could create closed-loop, self-regulating systems. Moreover, advances in 3D bioprinting are opening new avenues for customisation [157]. Stimuli-responsive bioinks can be used to fabricate dressings tailored to patient-specific wound geometry and pathology, supporting rapid, point-of-care production of therapeutic scaffolds. Ultimately, the future of intelligent wound dressings will depend not only on their biological responsiveness but also on their successful integration with digital health tools, scalable production, and clinically relevant design. Interdisciplinary collaboration among material scientists, engineers, clinicians, and regulators will be critical to translating these technologies from bench to bedside.

## Figures and Tables

**Table 1:** Summary of stimuli-responsive hydrogels: mechanisms, advantages, and applications in wound healing.

Stimulus	Response Mechanism	Functional Advantages	Applications
pH	pH-triggered structural change or degradation	Controlled release; infection reduction in acidic environment	Inflamed/infected wounds
Thermo	Sol–gel transition near LCST or UCST	Injectable; body-temp responsive; in situ gelling	Burns, deep wounds
ROS	ROS-induced degradation or release	Antioxidant delivery; oxidative stress reduction	Chronic wounds, diabetic ulcers
Enzyme	Enzymatic cleavage of crosslinks	Selective release; enzyme-responsive action	Diabetic wounds, chronic inflammation
Light	Photo-triggered conformational or thermal effect	Non-invasive control; light-activated therapy	Superficial wounds, PDT
Glucose	Glucose-sensitive degradation or release	Glucose-regulated delivery; diabetic wound care	Diabetic ulcers, insulin systems
Electro	Electrically induced ion flow or deformation	Remote control; promotes regeneration	Electrotherapy, nerve repair
Magnetic	Magnetic field-induced heating or motion	Localised activation; precise targeting	Deep-tissue healing, cancer therapy
DNA	DNA hybridisation or displacement	Programmable release; high specificity	Gene therapy, smart dressings

**Table 2:** Designs and mechanisms of different stimuli-responsive hydrogels for wound healing.

Stimulus Type	Hydrogel Material	Mechanism	Wound Type	Reference
pH	RSV-grafted CNF, PVA–borax	pH-triggered drug release via semi-IPN structure	Infected	[72]
	Nap-GFFKH, sodium alginate	Self-assembly under pH via microfluidic mixing	Infected	[131]
	PER-TBA, NaCl, CS	pH-cleavable Schiff base in dynamic network	General	[132]
	HA-ALD, ADH, N-CS	pH-degradable hydrazone and imine bonds enabling insulin release	Diabetic	[78]
	CMCS, 2-FPBA, EGCG	Dual pH-cleavable Schiff and borate bonding	Diabetic	[133]
	HA, AMP peptide (KK(SLKL)_3_KK)	pH-triggered AMP release via Schiff base crosslinking	Infected	[79]
	HAMA, GelMA-CA, Ag^+^	pH-triggered Ag^+^ release via polyphenol coordination	Acute	[134]
	Anthocyanin-based hydrogel	pH-indicated visible colour change for infection monitoring	Infected	[81]
Thermo	QCS, rGO-PDA, PNIPAM	LCST-induced sol–gel transition of PNIPAM	General	[135]
	Thermoresponsive chitosan (TCTS)	Thermo-induced phase behaviour enabling XDR bacterial suppression	Burn	[136]
	COL, GG, PNIPAM	Thermo/NIR-induced gelation for enhanced wound repair	General	[96]
	PNIPAM, HA	Thermo-induced contraction activates mechanotransduction for wound closure	Chronic	[105]
	PEG derivatives, CNT-OH	Thermoconductive PEG/CNT network enabling rapid heat dissipation	Burn	[137]
	Dihydromyricetin, CaO_2_NPs	Heat-softened structure for sustained delivery	Diabetic	[138]
Light	HA, EGF	UV-cleavable linker for EGF release	General	[139]
	CS, WS2, ciprofloxacin	NIR-induced photothermal gel activation	Infected	[140]
	Ag np, MOF, boronic acid, berberine	Visible-light-triggered ROS generation and synergistic photodynamic antibacterial activity	Infected	[122]
	Chitosan, AM NSs	NIR-triggered photothermal antibacterial activity via AM NSs	Infected	[123]
	Bi/MoS_2_	Synergistic PDT and PTT under NIR for enhanced antibacterial efficacy	Diabetic	[124]
	PAG, AA, GelMA, CuS, LAS	NIR-triggered heating via CuS for release	Diabetic	[141]
	GMH, PDA	UV-cleavable Schiff base network	Infected	[142]
ROS	EFM peptide hydrogel	ROS-triggered Met oxidation and gel breakdown	Diabetic	[48]
	PVA, TVA	ROS-cleavable phenylboronic ester linkage	Diabetic	[143]
	QCS, TA, HCl, NaHCO_3_	TA-based phenol ROS scavenging	Diabetic	[144]
	CQCS, PEG, hMnO_2_@PDA NPs	H_2_O_2_-responsive ROS scavenging via enzyme-mimetic MnO_2_ and antioxidant PDA	Infected	[128]
	PPBA, TA, PVA	ROS-cleavable boronic ester dynamic bonds	Diabetic	[145]
Enzyme	4-arm-PEG-MAL, MMP(W)x, PEG-SH, ADSC-exo	MMP-sensitive peptide cleavage	Diabetic	[115]
	A_9_K_2_ peptide, FBS, PAO, Cu^2+^, NaCl	LOX-triggered peptide hydrogelation	General	[146]
	Dual-layered hydrogel with AIE-PS and stem cell vesicles	Enzyme-triggered photosensitiser release and ROS-scavenging for staged repair	Burn	[147]
	Dex-TA / Dex-DG-TA	HRP-mediated enzymatic crosslinking	General	[148]

## Data Availability

No new data were created or analysed in this study.

## References

[1] Wang P., Cai F., Li Y., Yang X., Feng R., Lu H., Bai X., Han J. (2024). Emerging trends in the application of hydrogel-based biomaterials for enhanced wound healing: A literature review. Int. J. Biol. Macromol..

[2] Shariatzadeh F.J., Currie S., Logsetty S., Spiwak R., Liu S. (2024). Enhancing wound healing and minimizing scarring: A comprehensive review of nanofiber technology in wound dressings. Prog. Mater. Sci..

[3] Lindley L.E., Stojadinovic O., Pastar I., Tomic-Canic M. (2016). Biology and biomarkers for wound healing. Plast. Reconstr. Surg..

[4] Landén N.X., Li D., Ståhle M. (2016). Transition from inflammation to proliferation: A critical step during wound healing. Cell. Mol. Life Sci..

[5] Boodhoo K., Vlok M., Tabb D.L., Myburgh K.H., van de Vyver M. (2021). Dysregulated healing responses in diabetic wounds occur in the early stages postinjury. J. Mol. Endocrinol..

[6] Gomez-Florit M., Pardo A., Domingues R.M., Graça A.L., Babo P.S., Reis R.L., Gomes M.E. (2020). Natural-based hydrogels for tissue engineering applications. Molecules.

[7] Francesko A., Petkova P., Tzanov T. (2018). Hydrogel dressings for advanced wound management. Curr. Med. Chem..

[8] Chen C., Gu Y., Deng L., Han S., Sun X., Chen Y., Lu J.R., Xu H. (2014). Tuning gelation kinetics and mechanical rigidity of *β*-hairpin peptide hydrogels via hydrophobic amino acid substitutions. ACS Appl. Mater. Interfaces.

[9] Dong R., Guo B. (2021). Smart wound dressings for wound healing. Nano Today.

[10] Freedman B.R., Hwang C., Talbot S., Hibler B., Matoori S., Mooney D.J. (2023). Breakthrough treatments for accelerated wound healing. Sci. Adv..

[11] Hao Z., Li X., Zhang R., Zhang L. (2024). Stimuli-Responsive Hydrogels for Antibacterial Applications. Adv. Healthc. Mater..

[12] Deng Z., Yu R., Guo B. (2021). Stimuli-responsive conductive hydrogels: Design, properties, and applications. Mater. Chem. Front..

[13] Gounden V., Singh M. (2024). Hydrogels and wound healing: Current and future prospects. Gels.

[14] Liu J., Du C., Huang W., Lei Y. (2024). Injectable smart stimuli-responsive hydrogels: Pioneering advancements in biomedical applications. Biomater. Sci..

[15] Sun Z., Ding Y., Wang Z., Luo H., Feng Q., Cao X. (2024). Ros-responsive hydrogel with size-dependent sequential release effects for anti-bacterial and anti-inflammation in diabetic wound healing. Chem. Eng. J..

[16] Ding H., Tan P., Fu S., Tian X., Zhang H., Ma X., Gu Z., Luo K. (2022). Preparation and application of pH-responsive drug delivery systems. J. Control. Release.

[17] Zhou H., Zhu Y., Yang B., Huo Y., Yin Y., Jiang X., Ji W. (2024). Stimuli-responsive peptide hydrogels for biomedical applications. J. Mater. Chem. B.

[18] Xin H., Maruf D., Akin-Ige F., Amin S. (2024). Stimuli-responsive hydrogels for skin wound healing and regeneration. Emergent Mater..

[19] Alsaikhan F., Farhood B. (2024). Recent advances on chitosan/hyaluronic acid-based stimuli-responsive hydrogels and composites for cancer treatment: A comprehensive review. Int. J. Biol. Macromol..

[20] Chai Q., Jiao Y., Yu X. (2017). Hydrogels for biomedical applications: Their characteristics and the mechanisms behind them. Gels.

[21] Kumi M., Ejeromedoghene O., Sudane W.D., Zhang Z. (2024). Unlocking the biological response of smart Stimuli-Responsive hydrogels and their application in biological systems. Eur. Polym. J..

[22] Ejeromedoghene O., Omoniyi A.O., Akor E., Alowakennu M., Samson K.A., Abesa S., Zhang Z. (2024). Progress in stimuli-responsive hydrogel composites for digital technologies. Appl. Mater. Today.

[23] Zhang L., Zhou Y., Su D., Wu S., Zhou J., Chen J. (2021). Injectable, self-healing and pH responsive stem cell factor loaded collagen hydrogel as a dynamic bioadhesive dressing for diabetic wound repair. J. Mater. Chem. B.

[24] Liu F., Urban M.W. (2010). Recent advances and challenges in designing stimuli-responsive polymers. Prog. Polym. Sci..

[25] Li Z., Zhou Y., Li T., Zhang J., Tian H. (2022). Stimuli-responsive hydrogels: Fabrication and biomedical applications. View.

[26] Wang Z., Chen R., Yang S., Li S., Gao Z. (2022). Design and application of stimuli-responsive DNA hydrogels: A review. Mater. Today Bio.

[27] Barhoum A., Sadak O., Ramirez I.A., Iverson N. (2023). Stimuli-bioresponsive hydrogels as new generation materials for implantable, wearable, and disposable biosensors for medical diagnostics: Principles, opportunities, and challenges. Adv. Colloid Interface Sci..

[28] Zhang D., Ren B., Zhang Y., Xu L., Huang Q., He Y., Li X., Wu J., Yang J., Chen Q. (2020). From design to applications of stimuli-responsive hydrogel strain sensors. J. Mater. Chem. B.

[29] Khalid K.A., Nawi A.F.M., Zulkifli N., Barkat M.A., Hadi H. (2022). Aging and wound healing of the skin: A review of clinical and pathophysiological hallmarks. Life.

[30] Steed D.L. (1997). The role of growth factors in wound healing. Surg. Clin. N. Am..

[31] Peña O.A., Martin P. (2024). Cellular and molecular mechanisms of skin wound healing. Nat. Rev. Mol. Cell Biol..

[32] Cioce A., Cavani A., Cattani C., Scopelliti F. (2024). Role of the skin immune system in wound healing. Cells.

[33] Fabian T.C. (2007). Damage control in trauma: Laparotomy wound management acute to chronic. Surg. Clin..

[34] Choudhary V., Choudhary M., Bollag W.B. (2024). Exploring skin wound healing models and the impact of natural lipids on the healing process. Int. J. Mol. Sci..

[35] Raziyeva K., Kim Y., Zharkinbekov Z., Kassymbek K., Jimi S., Saparov A. (2021). Immunology of acute and chronic wound healing. Biomolecules.

[36] Martin P., Nunan R. (2015). Cellular and molecular mechanisms of repair in acute and chronic wound healing. Br. J. Dermatol..

[37] Tarnuzzer R.W., Schultz G.S. (1996). Biochemical analysis of acute and chronic wound environments. Wound Repair Regen..

[38] Monaco J.L., Lawrence W.T. (2003). Acute wound healing: An overview. Clin. Plast. Surg..

[39] Cañedo-Dorantes L., Cañedo-Ayala M. (2019). Skin acute wound healing: A comprehensive review. Int. J. Inflamm..

[40] Lawrence W.T. (1998). Physiology of the acute wound. Clin. Plast. Surg..

[41] Li J., Chen J., Kirsner R. (2007). Pathophysiology of acute wound healing. Clin. Dermatol..

[42] Yang Y., Zhong S., Meng F., Cui X. (2024). Multi-Functional hydrogels to promote diabetic wound Healing: A review. Chem. Eng. J..

[43] Morton L.M., Phillips T.J. (2016). Wound healing and treating wounds: Differential diagnosis and evaluation of chronic wounds. J. Am. Acad. Dermatol..

[44] Falanga V., Isseroff R.R., Soulika A.M., Romanelli M., Margolis D., Kapp S., Granick M., Harding K. (2022). Chronic wounds. Nat. Rev. Dis. Prim..

[45] Eriksson E., Liu P.Y., Schultz G.S., Martins-Green M.M., Tanaka R., Weir D., Gould L.J., Armstrong D.G., Gibbons G.W., Wolcott R. (2022). Chronic wounds: Treatment consensus. Wound Repair Regen..

[46] Li L., Wang Y., Huang Z., Xu Z., Cao R., Li J., Wu B., Lu J.R., Zhu H. (2023). An additive-free multifunctional *β*-glucan-peptide hydrogel participates in the whole process of bacterial-infected wound healing. J. Control. Release.

[47] Werdin F., Tenenhaus M., Rennekampff H.O. (2008). Chronic wound care. Lancet.

[48] Jia D., Li S., Jiang M., Lv Z., Wang H., Zheng Z. (2024). Facile reactive oxygen species-scavenging supramolecular hydrogel to promote diabetic wound healing. ACS Appl. Mater. Interfaces.

[49] Li S., Renick P., Senkowsky J., Nair A., Tang L. (2021). Diagnostics for wound infections. Adv. Wound Care.

[50] Cavallo I., Sivori F., Mastrofrancesco A., Abril E., Pontone M., Di Domenico E.G., Pimpinelli F. (2024). Bacterial biofilm in chronic wounds and possible therapeutic approaches. Biology.

[51] Versey Z., da Cruz Nizer W.S., Russell E., Zigic S., DeZeeuw K.G., Marek J.E., Overhage J., Cassol E. (2021). Biofilm-innate immune interface: Contribution to chronic wound formation. Front. Immunol..

[52] Darvishi S., Tavakoli S., Kharaziha M., Girault H.H., Kaminski C.F., Mela I. (2022). Advances in the sensing and treatment of wound biofilms. Angew. Chem. Int. Ed..

[53] Haidari H., Vasilev K., Cowin A., Kopecki Z. (2022). Bacteria-Activated Dual pH- and Temperature-Responsive Hydrogel for Targeted Elimination of Infection and Improved Wound Healing. ACS Appl. Mater. Interfaces.

[54] Guo J., Yao L., Wang X., Song R., Yang B., Jin D., Guo J., Wu G. (2024). Dual-Responsive Antibacterial Hydrogel Patch for Chronic-Infected Wound Healing. Biomacromolecules.

[55] Yang Y., Wang J., Huang S., Li M., Chen J., Pei D., Tang Z., Guo B. (2024). Bacteria-responsive programmed self-activating antibacterial hydrogel to remodel regeneration microenvironment for infected wound healing. Natl. Sci. Rev..

[56] Hurlow J., Wolcott R.D., Bowler P.G. (2025). Clinical management of chronic wound infections: The battle against biofilm. Wound Repair Regen..

[57] Alfei S., Schito G.C., Schito A.M., Zuccari G. (2024). Reactive oxygen species (ROS)-mediated antibacterial oxidative therapies: Available methods to generate ROS and a novel option proposal. Int. J. Mol. Sci..

[58] Gao D., Zhang Y., Bowers D.T., Liu W., Ma M. (2021). Functional hydrogels for diabetic wound management. APL Bioeng..

[59] Hu Y., Yu L., Dai Q., Hu X., Shen Y. (2024). Multifunctional antibacterial hydrogels for chronic wound management. Biomater. Sci..

[60] Chen Y., Wang X., Tao S., Wang Q., Ma P.Q., Li Z.B., Wu Y.L., Li D.W. (2023). Research advances in smart responsive-hydrogel dressings with potential clinical diabetic wound healing properties. Mil. Med. Res..

[61] Das I.J., Bal T. (2024). pH factors in chronic wound and pH-responsive polysaccharide-based hydrogel dressings. Int. J. Biol. Macromol..

[62] Fan Y., Wang H., Wang C., Xing Y., Liu S., Feng L., Zhang X., Chen J. (2024). Advances in smart-response hydrogels for skin wound repair. Polymers.

[63] Che X., Zhao T., Hu J., Yang K., Ma N., Li A., Sun Q., Ding C., Ding Q. (2024). Application of chitosan-based hydrogel in promoting wound healing: A review. Polymers.

[64] Singh J., Nayak P. (2023). pH-responsive polymers for drug delivery: Trends and opportunities. J. Polym. Sci..

[65] Liang Y., He J., Guo B. (2021). Functional hydrogels as wound dressing to enhance wound healing. ACS Nano.

[66] Lv X., Zhang J., Yang D., Shao J., Wang W., Zhang Q., Dong X. (2020). Recent advances in pH-responsive nanomaterials for anti-infective therapy. J. Mater. Chem. B.

[67] Han Z., Yuan M., Liu L., Zhang K., Zhao B., He B., Liang Y., Li F. (2023). pH-Responsive wound dressings: Advances and prospects. Nanoscale Horizons.

[68] Zhang W., Liu W., Long L., He S., Wang Z., Liu Y., Yang L., Chen N., Hu C., Wang Y. (2023). Responsive multifunctional hydrogels emulating the chronic wounds healing cascade for skin repair. J. Control. Release.

[69] Kizilel S., Cevik O. (2018). PH Responsive Hybrid Hydrogel and Method of Synthesis Thereof. U.S. Patent.

[70] Tulain U.R., Ahmad M., Rashid A., Malik M.Z., Iqbal F.M. (2018). Fabrication of Ph-responsive hydrogel and its in vitro and in vivo evaluation. Adv. Polym. Technol..

[71] Khan F., Atif M., Haseen M., Kamal S., Khan M.S., Shahid S., Nami S.A. (2022). Synthesis, classification and properties of hydrogels: Their applications in drug delivery and agriculture. J. Mater. Chem. B.

[72] Yang G., Zhang Z., Liu K., Ji X., Fatehi P., Chen J. (2022). A cellulose nanofibril-reinforced hydrogel with robust mechanical, self-healing, pH-responsive and antibacterial characteristics for wound dressing applications. J. Nanobiotech..

[73] Thambi T., Jung J.M., Lee D.S. (2023). Recent strategies to develop pH-sensitive injectable hydrogels. Biomater. Sci..

[74] Ren H., Zhang Z., Chen X., He C. (2024). Stimuli-Responsive Hydrogel Adhesives for Wound Closure and Tissue Regeneration. Macromol. Biosci..

[75] Tricou L.P., Al-Hawat M.L., Cherifi K., Manrique G., Freedman B.R., Matoori S. (2024). Wound pH-modulating strategies for diabetic wound healing. Adv. Wound Care.

[76] Jia X., Dou Z., Zhang Y., Li F., Xing B., Hu Z., Li X., Liu Z., Yang W., Liu Z. (2023). Smart responsive and controlled-release hydrogels for chronic wound treatment. Pharmaceutics.

[77] Psarrou M., Mitraki A., Vamvakaki M., Kokotidou C. (2023). Stimuli-responsive polysaccharide hydrogels and their composites for wound healing applications. Polymers.

[78] Li Z., Zhao Y., Liu H., Ren M., Wang Z., Wang X., Liu H., Feng Y., Lin Q., Wang C. (2021). pH-responsive hydrogel loaded with insulin as a bioactive dressing for enhancing diabetic wound healing. Mater. Des..

[79] Suo H., Hussain M., Wang H., Zhou N., Tao J., Jiang H., Zhu J. (2021). Injectable and pH-sensitive hyaluronic acid-based hydrogels with on-demand release of antimicrobial peptides for infected wound healing. Biomacromolecules.

[80] He J., Zhang Z., Yang Y., Ren F., Li J., Zhu S., Ma F., Wu R., Lv Y., He G. (2021). Injectable self-healing adhesive pH-responsive hydrogels accelerate gastric hemostasis and wound healing. Nano-Micro Lett..

[81] Huang D., Du J., Luo F., He G., Zou M., Wang Y., Lin Z., Wu D., Weng Z. (2024). Injectable Hydrogels with Integrated Ph Probes and Ultrasound-Responsive Microcapsules as Smart Wound Dressings for Visual Monitoring and On-Demand Treatment of Chronic Wounds. Adv. Healthc. Mater..

[82] Jin S., Mia R., Newton M.A.A., Cheng H., Gao W., Zheng Y., Dai Z., Zhu J. (2024). Nanofiber-reinforced self-healing polysaccharide-based hydrogel dressings for pH discoloration monitoring and treatment of infected wounds. Carbohydr. Polym..

[83] Knopf G.K., Sinar D. (2017). Flexible hydrogel actuated graphene-cellulose biosensor for monitoring pH. Proceedings of the 2017 IEEE International Symposium on Circuits and Systems (ISCAS).

[84] Tymetska S., Shymborska Y., Stetsyshyn Y., Budkowski A., Bernasik A., Awsiuk K., Donchak V., Raczkowska J. (2023). Thermoresponsive smart copolymer coatings based on P (NIPAM-co-HEMA) and P (OEGMA-co-HEMA) brushes for regenerative medicine. ACS Biomater. Sci. Eng..

[85] Said S.S., Campbell S., Hoare T. (2019). Externally addressable smart drug delivery vehicles: Current technologies and future directions. Chem. Mater..

[86] Abbasi A.R., Sohail M., Minhas M., Khaliq T., Kousar M., Khan S., Hussain Z., Munir A. (2020). Bioinspired sodium alginate based thermosensitive hydrogel membranes for accelerated wound healing. Int. J. Biol. Macromol..

[87] Yu Y., Cheng Y., Tong J., Zhang L., Wei Y., Tian M. (2021). Recent advances in thermo-sensitive hydrogels for drug delivery. J. Mater. Chem. B.

[88] Gu R., Zhou H., Zhang Z., Lv Y., Pan Y., Li Q., Shi C., Wang Y., Wei L. (2023). Research progress related to thermosensitive hydrogel dressings in wound healing: A review. Nanoscale Adv..

[89] Khan B., Arbab A., Khan S., Fatima H., Bibi I., Chowdhry N.P., Ansari A.Q., Ursani A.A., Kumar S., Hussain J. (2023). Recent progress in thermosensitive hydrogels and their applications in drug delivery area. MedComm–Biomater. Appl..

[90] Castillo-Henríquez L., Castro-Alpízar J., Lopretti-Correa M., Vega-Baudrit J. (2021). Exploration of bioengineered scaffolds composed of thermo-responsive polymers for drug delivery in wound healing. Int. J. Mol. Sci..

[91] Luo J., Zhao X., Guo B., Han Y. (2023). Preparation, thermal response mechanisms and biomedical applications of thermosensitive hydrogels for drug delivery. Expert Opin. Drug Deliv..

[92] Markandeywar T.S., Singh D., Narang R.K. (2024). A Complete sojorum on thermosensitive hydrogels for wound healing: Recent developments and ongoing research. Curr. Drug Ther..

[93] Tian M.L., Zhou J.F., Qi X., Shen R. (2021). Thermo-sensitive hydrogel and their biomedical applications. IOP Conf. Ser. Earth Environ. Sci..

[94] Cao M., Wang Y., Hu X., Gong H., Li R., Cox H., Zhang J., Waigh T.A., Xu H., Lu J.R. (2019). Reversible thermoresponsive peptide–PNIPAM hydrogels for controlled drug delivery. Biomacromolecules.

[95] Lacroce E., Rossi F. (2022). Polymer-based thermoresponsive hydrogels for controlled drug delivery. Expert Opin. Drug Deliv..

[96] Zhang M., Deng F., Tang L., Wu H., Ni Y., Chen L., Huang L., Hu X., Lin S., Ding C. (2021). Super-ductile, injectable, fast self-healing collagen-based hydrogels with multi-responsive and accelerated wound-repair properties. Chem. Eng. J..

[97] Feng T., Wu H., Ma W., Wang Z., Wang C., Wang Y., Wang S., Zhang M., Hao L. (2022). An injectable thermosensitive hydrogel with a self-assembled peptide coupled with an antimicrobial peptide for enhanced wound healing. J. Mater. Chem. B.

[98] Safakas K., Saravanou S.F., Iatridi Z., Tsitsilianis C. (2021). Alginate-g-PNIPAM-based thermo/shear-responsive injectable hydrogels: Tailoring the rheological properties by adjusting the LCST of the grafting chains. Int. J. Mol. Sci..

[99] Bonetti L., De Nardo L., Farè S. (2021). Thermo-responsive methylcellulose hydrogels: From design to applications as smart biomaterials. TIssue Eng. Part B Rev..

[100] Tang S., Gong Z., Wang Z., Gao X., Zhang X. (2022). Multifunctional hydrogels for wound dressings using xanthan gum and polyacrylamide. Int. J. Biol. Macromol..

[101] Sekar M.P., Budharaju H., Sethuraman S., Sundaramurthi D. (2023). Carboxymethyl cellulose-agarose-gelatin: A thermoresponsive triad bioink composition to fabricate volumetric soft tissue constructs. SLAS Technol..

[102] Zhang S., Ge G., Qin Y., Li W., Dong J., Mei J., Ma R., Zhang X., Bai J., Zhu C. (2022). Recent advances in responsive hydrogels for diabetic wound healing. Mater. Today Bio.

[103] Xu X., Wang Y., Han C.G., Lin J., Shen Q., Lan Y., Long L., Tan X., Liu J., Liu S. (2024). Poison Turned Panacea: Arsenic Trioxide Loaded Hydrogel for Inhibiting Scar Formation in Wound Healing. ACS Biomater. Sci. Eng..

[104] Sangboonruang S., Semakul N., Manokruang K., Khammata N., Jantakee K., Mai-ngam K., Charoenla S., Khamnoi P., Saengsawang K., Wattananandkul U. (2024). Multifunctional poloxamer-based thermo-responsive hydrogel loaded with human lactoferricin niosomes: In vitro study on anti-bacterial activity, accelerate wound healing, and anti-inflammation. Int. J. Pharm. X.

[105] Zhao Y., Yi B., Hu J., Zhang D., Li G., Lu Y., Zhou Q. (2023). Double Cross-Linked Biomimetic Hyaluronic Acid-Based Hydrogels with Thermo-Stimulated Self-Contraction and Tissue Adhesiveness for Accelerating Post-Wound Closure and Wound Healing. Adv. Funct. Mater..

[106] Li Z., Huang J., Jiang Y., Liu Y., Qu G., Chen K., Zhao Y., Wang P., Wu X., Ren J. (2022). Novel Temperature-Sensitive Hydrogel Promotes Wound Healing Through YAP and MEK-Mediated Mechanosensitivity. Adv. Healthc. Mater..

[107] Elbediwy A., Vincent-Mistiaen Z.I., Spencer-Dene B., Stone R., Boeing S., Wculek S., Cordero J.B., Tan E., Ridgway R., Brunton V. (2016). Integrin signalling regulates YAP and TAZ to control skin homeostasis. Development.

[108] Wang X., Wang Q. (2021). Enzyme-laden bioactive hydrogel for biocatalytic monitoring and regulation. Accounts Chem. Res..

[109] Li P., Zhong Y., Wang X., Hao J. (2020). Enzyme-regulated healable polymeric hydrogels. ACS Cent. Sci..

[110] Hu J., Zhang G., Liu S. (2012). Enzyme-responsive polymeric assemblies, nanoparticles and hydrogels. Chem. Soc. Rev..

[111] Minehan R.L., Del Borgo M.P. (2022). Controlled release of therapeutics from enzyme-responsive biomaterials. Front. Biomater. Sci..

[112] Khattak S., Ullah I., Sohail M., Akbar M.U., Rauf M.A., Ullah S., Shen J., Xu H.T. (2025). Endogenous/exogenous stimuli-responsive smart hydrogels for diabetic wound healing. Aggregate.

[113] Guo S., Zhang Q., Li X., Wang Q., Li X., Wang P., Xue Q. (2025). Bacterial-responsive biodegradable silver nanoclusters composite hydrogel for infected wound therapy. Colloids Surfaces B Biointerfaces.

[114] Distler T., McDonald K., Heid S., Karakaya E., Detsch R., Boccaccini A.R. (2020). Ionically and enzymatically dual cross-linked oxidized alginate gelatin hydrogels with tunable stiffness and degradation behavior for tissue engineering. ACS Biomater. Sci. Eng..

[115] Jiang T., Liu S., Wu Z., Li Q., Ren S., Chen J., Xu X., Wang C., Lu C., Yang X. (2022). ADSC-exo@ MMP-PEG smart hydrogel promotes diabetic wound healing by optimizing cellular functions and relieving oxidative stress. Mater. Today Bio.

[116] Li N., Zhan A., Jiang Y., Liu H. (2022). A novel matrix metalloproteinases-cleavable hydrogel loading deferoxamine accelerates diabetic wound healing. Int. J. Biol. Macromol..

[117] Zhou W., Duan Z., Zhao J., Fu R., Zhu C., Fan D. (2022). Glucose and MMP-9 dual-responsive hydrogel with temperature sensitive self-adaptive shape and controlled drug release accelerates diabetic wound healing. Bioact. Mater..

[118] Sobczak M. (2022). Enzyme-responsive hydrogels as potential drug delivery systems—state of knowledge and future prospects. Int. J. Mol. Sci..

[119] Wang M., Gao B., Wang X., Li W., Feng Y. (2022). Enzyme-responsive strategy as a prospective cue to construct intelligent biomaterials for disease diagnosis and therapy. Biomater. Sci..

[120] Maleki A., He J., Bochani S., Nosrati V., Shahbazi M.A., Guo B. (2021). Multifunctional photoactive hydrogels for wound healing acceleration. ACS Nano.

[121] Xu Y., Chen H., Fang Y., Wu J. (2022). Hydrogel combined with phototherapy in wound healing. Adv. Healthc. Mater..

[122] He Q.T., Qian P., Yang X.Y., Kuang Q., Lin Y.T., Yi W., Tian T., Cai Y.P., Hong X.J. (2024). Rational design of bacteria-targeted and photo-responsive MOF gel with antibacterial and anti-inflammatory function for infected wound healing. Chem. Eng. J..

[123] Liu Y., Xiao Y., Cao Y., Guo Z., Li F., Wang L. (2020). Construction of chitosan-based hydrogel incorporated with antimonene nanosheets for rapid capture and elimination of bacteria. Adv. Funct. Mater..

[124] Liu C., Li Y., Li W., Fan Y., Zhou W., Xiao C., Yu P., Liu Y., Liu X., Huang Z. (2024). LSPR-enhanced photoresponsive antibacterial efficiency of Bi/MoS2-loaded fibrin gel for management of diabetic wounds. Int. J. Biol. Macromol..

[125] He Z., Xu Q., Newland B., Foley R., Lara-Sáez I., Curtin J.F., Wang W. (2021). Reactive oxygen species (ROS): Utilizing injectable antioxidative hydrogels and ROS-producing therapies to manage the double-edged sword. J. Mater. Chem. B.

[126] Zhang Y., Xu Y., Hu W., Ma X., Hu J., Ye Y., Yang S., Yu Y., Li N., Zheng D. (2025). An adhesive and self-healing ROS-scavenging hydrogel loading with hMSC-derived exosomes for diabetic wound healing. Int. J. Pharm..

[127] Huang C., Dong L., Zhao B., Lu Y., Huang S., Yuan Z., Luo G., Xu Y., Qian W. (2022). Anti-inflammatory hydrogel dressings and skin wound healing. Clin. Transl. Med..

[128] Wang Y., Chen C., He C., Dong W., Yang X., Kong Q., Yan B., He J. (2025). Quaternized chitosan-based biomimetic nanozyme hydrogels with ROS scavenging, oxygen generating, and antibacterial capabilities for diabetic wound repair. Carbohydr. Polym..

[129] Pan X.y., Wang Z.h., Wu X.q., Guo C.r., Yang L.x., Liu H.r., Wang Y.h., Chen W.j., Wang J.j., Nan K.h. (2025). ROS scavenging and corneal epithelial wound healing by a self-crosslinked tissue-adhesive hydrogel based-on dual-functionalized hyaluronic acid. Int. J. Biol. Macromol..

[130] Dong Y., Wang Z. (2023). ROS-scavenging materials for skin wound healing: Advancements and applications. Front. Bioeng. Biotechnol..

[131] Cui T., Li X., He S., Xu D., Yin L., Huang X., Deng S., Yue W., Zhong W. (2020). Instant self-assembly peptide hydrogel encapsulation with fibrous alginate by microfluidics for infected wound healing. ACS Biomater. Sci. Eng..

[132] Fan L., He Z., Peng X., Xie J., Su F., Wei D.X., Zheng Y., Yao D. (2021). Injectable, intrinsically antibacterial conductive hydrogels with self-healing and pH stimulus responsiveness for epidermal sensors and wound healing. ACS Appl. Mater. Interfaces.

[133] Su K., Deng D., Wu X., Song Y., Sun Y., Wang X., Zhang Z., Li J., Yan Z., Shang X. (2024). On-demand detachable adhesive hydrogel based on dual dynamic covalent cross-linked with NIR/pH dual-responsive properties for diabetic wound healing. Chem. Eng. J..

[134] Gui Q., Ding N., Wu H., Liu J., Geng Y., Zhu J., Gao M., Du A., Yue B., Zhu L. (2024). Development of a pH-Responsive Antimicrobial and Potent Antioxidant Hydrogel for Accelerated Wound Healing: A Game Changer in Drug Delivery. Adv. Biol..

[135] Li M., Liang Y., He J., Zhang H., Guo B. (2020). Two-pronged strategy of biomechanically active and biochemically multifunctional hydrogel wound dressing to accelerate wound closure and wound healing. Chem. Mater..

[136] Ahovan Z.A., Khosravimelal S., Eftekhari B., Mehrabi S., Hashemi A., Eftekhari S., Milan P.B., Mobaraki M., Seifalian A., Gholipourmalekabadi M. (2020). Thermo-responsive chitosan hydrogel for healing of full-thickness wounds infected with XDR bacteria isolated from burn patients: In vitro and in vivo animal model. Int. J. Biol. Macromol..

[137] Shi W., Song N., Huang Y., He C., Zhang M., Zhao W., Zhao C. (2022). Improved Cooling Performance of Hydrogel Wound Dressings via Integrating Thermal Conductivity and Heat Storage Capacity for Burn Therapy. Biomacromolecules.

[138] Liu X., Ding Q., Liu W., Zhang S., Wang N., Chai G., Wang Y., Sun S., Zheng R., Zhao Y. (2024). A Poloxamer 407/chitosan-based thermosensitive hydrogel dressing for diabetic wound healing via oxygen production and dihydromyricetin release. Int. J. Biol. Macromol..

[139] Lim S., Kim J.A., Chun Y.H., Lee H.J. (2023). Hyaluronic acid hydrogel for controlled release of heterobifunctional photocleavable linker-modified epidermal growth factor in wound healing. Int. J. Biol. Macromol..

[140] Yang N., Zhu M., Xu G., Liu N., Yu C. (2020). A near-infrared light-responsive multifunctional nanocomposite hydrogel for efficient and synergistic antibacterial wound therapy and healing promotion. J. Mater. Chem. B.

[141] He D., Liao C., Li P., Liao X., Zhang S. (2024). Multifunctional photothermally responsive hydrogel as an effective whole-process management platform to accelerate chronic diabetic wound healing. Acta Biomater..

[142] Wei Y., Fu J., Liu E., Gao J., Lv Y., Li Z. (2024). Injectable hydrogels doped with PDA nanoparticles for photothermal bacterial inhibition and rapid wound healing in vitro. RSC Adv..

[143] Zhao H., Huang J., Li Y., Lv X., Zhou H., Wang H., Xu Y., Wang C., Wang J., Liu Z. (2020). ROS-scavenging hydrogel to promote healing of bacteria infected diabetic wounds. Biomaterials.

[144] Pan W., Qi X., Xiang Y., You S., Cai E., Gao T., Tong X., Hu R., Shen J., Deng H. (2022). Facile formation of injectable quaternized chitosan/tannic acid hydrogels with antibacterial and ROS scavenging capabilities for diabetic wound healing. Int. J. Biol. Macromol..

[145] Ni Z., Yu H., Wang L., Huang Y., Lu H., Zhou H., Liu Q. (2022). Multistage ROS-responsive and natural polyphenol-driven prodrug hydrogels for diabetic wound healing. ACS Appl. Mater. Interfaces.

[146] Bai J., Chen C., Wang J., Zhang Y., Cox H., Zhang J., Wang Y., Penny J., Waigh T., Lu J.R. (2016). Enzymatic regulation of self-assembling peptide A9K2 nanostructures and hydrogelation with highly selective antibacterial activities. ACS Appl. Mater. Interfaces.

[147] Zhao M., Kang M., Wang J., Yang R., Zhong X., Xie Q., Zhou S., Zhang Z., Zheng J., Zhang Y. (2024). Stem Cell-Derived Nanovesicles Embedded in Dual-Layered Hydrogel for Programmed ROS Regulation and Comprehensive Tissue Regeneration in Burn Wound Healing. Adv. Mater..

[148] Jin R., Hiemstra C., Zhong Z., Feijen J. (2007). Enzyme-mediated fast in situ formation of hydrogels from dextran–tyramine conjugates. Biomaterials.

[149] Wu Y., Wang Y., Long L., Hu C., Kong Q., Wang Y. (2022). A spatiotemporal release platform based on pH/ROS stimuli-responsive hydrogel in wound repairing. J. Control. Release.

[150] Zhang Y., Gao X., Tang X., Peng L., Zhang H., Zhang S., Hu Q., Li J. (2023). A dual pH-and temperature-responsive hydrogel produced in situ crosslinking of cyclodextrin-cellulose for wound healing. Int. J. Biol. Macromol..

[151] Tian R., Liu J., Dou G., Lin B., Chen J., Yang G., Li P., Liu S., Jin Y., Qiu X. (2022). Synergistic antibiosis with spatiotemporal controllability based on multiple-responsive hydrogel for infectious cutaneous wound healing. Smart Mater. Med..

[152] Wu J., Xue W., Yun Z., Liu Q., Sun X. (2024). Biomedical applications of stimuli-responsive “smart” interpenetrating polymer network hydrogels. Mater. Today Bio.

[153] Brumberg V.A., Bikmulina P.Y., Pozdnyakov A.A., Heydari Z., Zimulkina D.R., Smirnova O.A., de Sena Pereira F.D.A., Nesterova A.M., Kotova S.L., Vosough M. (2025). Scaling liver bioprinting: A guide for usage of the hepatic extracellular matrix as a bioink. Int. J. Bioprinting.

[154] Moura D., Rohringer S., Ferreira H.P., Pereira A.T., Barrias C., Magalhães F.D., Bergmeister H., Gonçalves I.C. (2023). Long-term in vivo degradation and biocompatibility of degradable pHEMA hydrogels containing graphene oxide. Acta Biomater..

[155] Li Y., Saiding Q., Wang Z., Cui W. (2023). Engineered Biomimetic Hydrogels for Organoids. Prog. Mater. Sci..

[156] Neumann M., di Marco G., Iudin D., Viola M., van Nostrum C., van Ravensteijn B.G.P., Vermonden T. (2023). Stimuli-Responsive Hydrogels: The Dynamic Smart Biomaterials of Tomorrow. Macromolecules.

[157] El-Husseiny H.M., Mady E.A., Hamabe L., Abugomaa A., Shimada K., Yoshida T., Tanaka T., Yokoi A., Elbadawy M., Tanaka R. (2021). Smart/stimuli-responsive hydrogels: Cutting-edge platforms for tissue engineering and other biomedical applications. Mater. Today Bio.

